# Could selected probiotics have beneficial effects on clinical outcome of severe traumatic brain injury patients?

**DOI:** 10.1186/cc13662

**Published:** 2014-03-17

**Authors:** D Pavelescu, L Mirea, I Grintescu

**Affiliations:** 1 Emergency Hospital Floreasca, Bucharest, Romania

## Introduction

Severe traumatic brain injury (TBI) is a major cause of death in people between 19 and 45 years old. Gastrointestinal dysfunction is the most common complication due to mucosal ischemia, motility disorders, and disruption of the gut barrier, with severe consequences: malnutrition, weight loss, and high risk of infections [1]. Therefore, maintaining the intestinal barrier function is a systematic engineering project. Selected new probiotics due to the capacity to bind and neutralize toxins, and to interfere with pathogen adherence, by immunomodulatory properties, mopping up the infection, could improve recovery of critically ill patients [2]. Our aim was to assess the effects of a new probiotic in an early enteral regimen on clinical outcome of severe TBI patients, in terms of VAP incidence, tolerance to enteral nutrition, duration of mechanical ventilation, and mortality rate.

## Methods

A prospective randomized 1-year study of 64 patients 19 to 78 years old allocated to receive for 10 days either an early enteral diet plus a new probiotic (Bioent; *Lactobacillus bulgaricus *10 trillion CFU/cp + activated charcoal) every 6 hours (Group A) or the same formula without probiotics (Group B). The diets were isocaloric and isonitrogenous, and there were no differences between groups in gender, age, and nutritional status. We assessed the VAP incidence, duration of mechanical ventilation, tolerance to enteral nutrition, length of ICU stay, duration of diarrhea episodes, and mortality rate. The ANOVA test and *t *test were carried out; *P *< 0.05 was considered significant.

## Results

**Figure 1:**
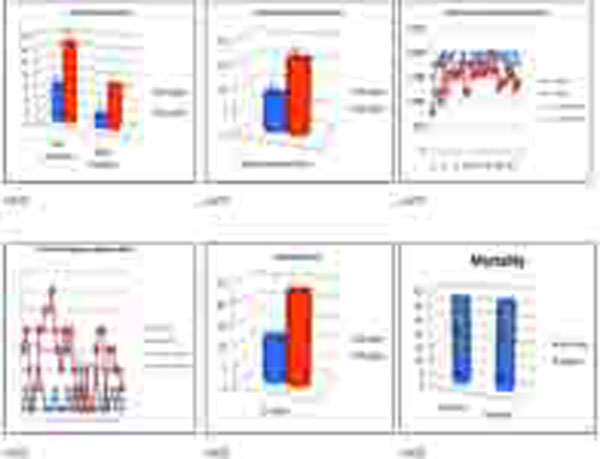


The infection rate was higher in group B, the duration of mechanical ventilation was shorter in group A, and the patients in group A received 91.7% of total caloric needs by an enteral route versus 74.68% in group B. There is a significant difference in the number of diarrhea episodes, and the ICU length of stay was significantly lower in group A; there was no significant difference in the mortality rate between groups. See Figure 1.

## Conclusion

Early administration of new probiotics to severe TBI patients could have beneficial effects in terms of reduction of GI dysfunction, VAP incidence and length of ICU stay.
